# Investigation of Phonon Scattering on the Tunable Mechanisms of Terahertz Graphene Metamaterials

**DOI:** 10.3390/nano10010039

**Published:** 2019-12-23

**Authors:** Xiaoyong He, Fangting Lin, Feng Liu, Hao Zhang

**Affiliations:** 1 Department of Physics, Mathematics & Science College, Shanghai Normal University, No. 100 Guilin Road, Shanghai 200234, China; nounou7@163.com (F.L.);; 2 Shanghai Key Lab for Astrophysics, No. 100 Guilin Road, Shanghai 200234, China

**Keywords:** terahertz, graphene, metamaterials, phonon scatterings

## Abstract

The influences of different kinds of phonon scatterings (i.e., acoustic (AC) phonon, impurity, and longitudinal optical (LO) phonon scatterings) on the tunable propagation properties of graphene metamaterials structures have been investigated, also including the effects of graphene pattern structures, Fermi levels, and operation frequencies. The results manifested that, at room temperature, AC phonon scattering dominated, while with the increase in temperature, the LO phonon scattering increased significantly and played a dominate role if temperature goes beyond 600 K. Due to the phonon scatterings, the resonant properties of the graphene metamaterial structure indicated an optimum value (about 0.5–0.8 eV) with the increase in Fermi level, which were different from the existing results. The results are very helpful to understand the tunable mechanisms of graphene functional devices, sensors, modulators, and antennas.

## 1. Introduction

The terahertz (THz) frequency range lies in the microwave and infrared wavelength ranges of the electromagnetic spectrum, showing potential applications in the practical fields of spectral analysis, imaging, homeland security detection, and high-speed wireless communication (in the range of several hundred Gbit s^−1^) [1,2,3,4,5,6,7,8]. But to further substantial development of THz science and technology, there is a high demand for well-performing functional waveguide devices and components. Because of relatively long wavelengths and the scarcity of natural materials that interact strongly with THz waves, the manipulation of THz waves is still a challenging task. Artificially designed subwavelength unit cells (meta-molecules) [9] and the emergence of metamaterials (MMs) were capable of exotic properties and helpful to overcome this dilemma [10,11,12]. Presently, the key disadvantages of plasmonic and MM devices were a lack of tunability of the resonant frequency and large distillation losses. Two-dimensional (2D) materials, such as black phosphorus, transition metal molybdenum disulfide, topological insulators (Bi_2_Ti_3_), and graphene [13,14,15,16], provided good alternatives for the exploration of novel flexible functional devices. Especially, with the merits of large carrier mobility and its strong interaction with light and pattern structures, as well as versatile tenability [17,18,19,20,21,22], graphene membrane attracted significant interest and have been functionalized as a playground to design novel plasmonic MM devices [23,24,25,26].

Graphene surface plasmons, i.e., the collective oscillations of free Dirac charge carriers coupled to electromagnetic fields, stimulated the propagation modes with extreme subwavelength confinement and relatively larger propagation lengths [27,28,29]. Recently, much research work was devoted to exploring graphene devices, e.g., optical modulators, photo-detectors, antennas, and biosensors [30,31,32]. On the base of the hetero-structures of graphene/h-BN/metal, Lundeberg et al. experimentally investigated the tunable nonlocal quantum effects in graphene plasmonic systems by using the near-field imaging experimental systems [33]. Because the conductivity and plasmonic properties of the graphene layer were closely associated with phonon scattering, some research has been carried out to investigate their effects on device performances. For instance, by using the graphene nanoribbons, Yan et al. experimentally studied the damping mechanisms of graphene plasmons, indicating that the plasmon lifetime and quality factors were limited by the optical phonons and surface polar phonons in the polar substrate layer [1]. Including the effects of acoustic (AC) phonon scattering, impurity phonon scattering and substrate phonons scattering, Low et al. compared the dispersion and propagation properties of the plasmon polaritons on the hetero-structures of 2D thin membrane/dielectric layers, which manifested good mode confinement and a better figure of merits than those of metal thin layers [16]. Based on the split ring resonators (SRRs) structure, Shen et al. discussed the scattering time and resistance on the graphene MM structure, displaying that if the dissipation losses were large, the low frequency inductance-capacitance (LC) resonance was very weak, but due to the large kinetic inductance of the graphene layer, the high frequency electric dipolar resonance was strong [34]. By exploiting the near-field imaging technique, the dispersion and damping properties of graphene membrane were investigated on the base of the graphene/*h*-BN structure, revealing that the damping losses of graphene surface plasmons (SPs) were closely depended on the thermal phonons in graphene and *h*-BN substrates [35]. 

In most of the published articles about tunable graphene functional devices [1,29,30,31,32], the graphene conductivities were obtained on assumption that the scattering rate was a constant, which was phenomenological in nature and not involved the microscopic origin of phonon scattering. This constant scattering rate model failed to provide adequate accuracy in the presence of inelastic scattering processes. There was a large difference between the experiments and simulation results. For instance, in References [29,30] the resonant curves of the experimental results were broader than those of the simulation results, possibly coming from the fact that phonon scatterings were not taken into account. Actually, besides Fermi levels and temperatures, the graphene conductivities were also closely related to phonon scatterings, which reduced the carrier motilities, just like most of the semiconductors [36,37,38]. As an interesting research topic, to investigate the phonon scatterings and relaxation losses of a graphene layer was fundamentally important to study the tunable mechanisms. Regretfully, there were little reports in this aspect. Therefore, the scattering mechanisms of different kinds of phonon scatterings, such as acoustic (AC) phonon, impurity and longitudinal optical (LO) phonon scatterings are discussed. Additionally, as examples, the effects of phonon scatterings on the tunable properties of graphene MM structures are also discussed. The results manifested that, at room temperature, the AC phonon scattering dominated the conductivity, while LO phonon scattering increased drastically with temperature and Fermi level, and played a dominating role above 600 K. For the graphene pattern structures, if the effects of phonon scatterings were considered, the resonant properties showed a peak with the increase in Fermi level. The Supplementary File shows the fabrication, transfer, and characterization of graphene metamaterials structures.

## 2. Research Methods

As a typical 2D membrane, the complex conductivity of graphene was associated with the operation frequency *ω*, chemical potential *μ_c_*, the environmental temperature *T*, and the relaxation time *τ*. Under the random phase approximation, the graphene conductivity was described as [39]

(1) $$\begin{array}{ll} \sigma_{g}= & \frac{j}{\omega +j/\tau }\frac{e^{2}2k_{B}T}{\pi \hbar^{2}}\ln\left[2\cosh\frac{\mu_{c}}{2k_{B}T}\right]\\ & +\frac{e^{2}}{4\hbar^{2}}\left[G\left(\frac{\hbar \omega }{2}\right)+j\frac{4\hbar \omega }{\pi }\int_{0}^{\infty }\frac{G\left(\xi \right)-G\left(\hbar \omega /2\right)}{{\left(\hbar \omega \right)}^{2}-4\xi^{2}}d\xi \right] \end{array}$$

(2) $$G\left(\xi \right)=\frac{\sinh\left(\xi /k_{B}T\right)}{\cosh\left(\xi /k_{B}T\right)+\cosh\left(\mu_{c}/k_{B}T\right)},$$

where *j* is the imaginary unit, *ω* is the operation frequency of incident light, *k_B_* is Boltzmann’s constant, *ℏ* is the reduced Planck’s constant, *μ_c_* is the chemical potential (Fermi level *E*_f_), and the relaxation time is τ. The graphene permittivity was expressed as

(3) $$\varepsilon_{g}=1+j\frac{\sigma_{g}}{\omega \varepsilon_{0}\Delta }$$

where Δ is the graphene layer thickness and *ɛ*_0_ is the permittivity of free space.

In many published articles, the scattering time was assumed as a constant, about 0.1–1.0 *ps* [34]. But the influences of phonon scattering were important at a large carrier concentration and Fermi level. Based on the Boltzmann transport theory, the graphene conductivity was given by [40]:

(4) $$\sigma =e^{2}D(E_{F})\frac{v_{F}^{2}}{2}\langle \tau \rangle$$

where *v_F_* is the Fermi velocity, $D\left(E_{F}\right)=(g_{s}g_{v}/2\pi \hbar ²)E_{F}/v_{F}^{2}$ is the density of state, and *v*_F_ ≈ 10⁶ m/s, <τ> is the relaxation time averaged over energy.

(5) $$\langle \tau \rangle =\frac{\int d\varepsilon D(\varepsilon )\tau \left(\varepsilon \right)\left[-\frac{df\left(\varepsilon \right)}{d\varepsilon }\right]}{\int d\varepsilon D(\varepsilon )\left[-\frac{df\left(\varepsilon \right)}{d\varepsilon }\right]},$$

in which *f(ɛ)* is the Fermi distribution function.

The scattering mechanisms in the graphene layer mainly included acoustic phonon scattering, impurity scattering, and optical phonon scattering. For longitudinal acoustic phonons, the energy dependent relaxation time *τ_ac_*(*ɛ*_k_) is expressed as

(6) $$\tau_{ac}^{-1}\left(\varepsilon_{k}\right)=\frac{1}{\hbar^{3}}\frac{k_{B}T}{4v_{F}^{2}}\frac{D_{A}^{2}}{\rho v_{ph}^{2}}\varepsilon_{k},$$

where *D*_A_ = 18 eV is the acoustic deformation potential, *ρ* = 7.6 × 10^−7^ kg/m^2^ is the graphene density, and *v*_ph_ = 2 × 10^4^ m/s is the phonon velocity of the longitudinal acoustic mode.

The scattering rate arising from the charged impurity scattering was derived as [41]

(7) $$\tau_{imp}^{-1}\left(\varepsilon_{k}\right)=\frac{n_{i}}{4\pi \hbar^{3}}\frac{\varepsilon_{k}}{v_{F}^{2}}\int_{}^{}d\theta \frac{V^{2}}{{\left(1+q_{s}/q\right)}^{2}}\left(1-\cos^{2}\theta \right)$$

where *n*_i_ is the impurity density, *q* is the scattering wave vector, *q*_s_ is the Thomas–Fermi screening wave vector, *q*_s_ = *e*^2^*D*(*E*_F_)/2ɛ_avg_, *D*(*E*_F_) is the density of states at Fermi energy, *V* = *e*^2^/2ɛ_avg_, *q* is the Fourier transform of the 2D potential energy, *q* = 2*k*sin(*θ*/2), where *k* is the wave vector and *θ* the scattering angle.

The optical phonon scattering rates can be written as [42]

(8) $$\tau_{op}^{-1}\left(\varepsilon_{k}\right)=\frac{D_{0}^{2}}{2\hbar^{2}v_{F}^{2}\rho \omega_{0}}n_{op}\left(E_{F}+\hbar \omega_{0}\right),$$

where *D*_0_ = 5 × 10⁹ eV/cm is the deformation potential for the longitudinal optical phonons, *n*_op_ = (e^ℏω^^_0_/*k*^_B_*^T^*− 1)^−1^ is the optical phonon number, and *ℏω_0_* is the optical phonon energy.

The total scattering rates as a function of the carrier energy can be written as

(9) $$\tau_{\mathrm{total}}^{-1}\left(\varepsilon_{k}\right)=\tau_{\mathrm{ac}}^{-1}\left(\varepsilon_{k}\right)+\tau_{\mathrm{imp}}^{-1}\left(\varepsilon_{k}\right)+\tau_{\mathrm{op}}^{-1}\left(\varepsilon_{k}\right).$$

The relaxation time *τ* was related to mobility *μ_DC_* with the following equations

(10) $$\tau =\mu_{DC}E_{f}/ev_{F}^{2}.$$

## 3. Results and Discussion

**Figure 1:**
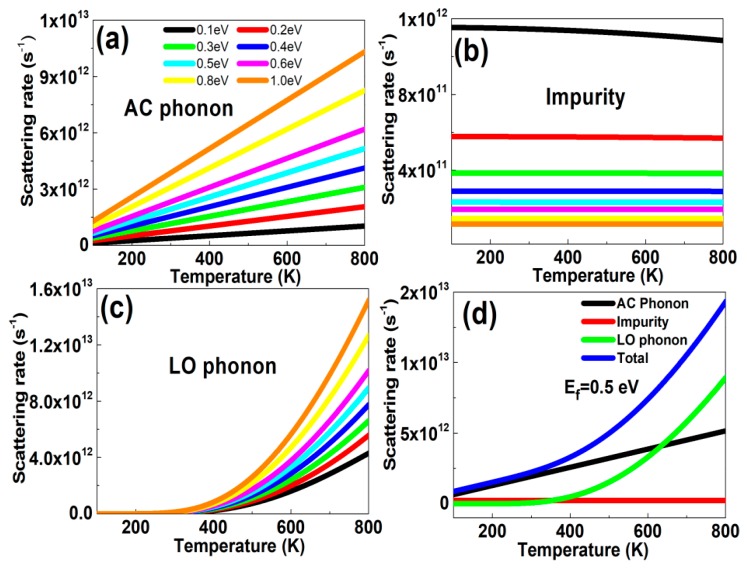
The scattering rates versus temperatures at different Fermi levels. (**a**) Acoustic (AC) phonon scattering, (**b**) impurity scattering, and (**c**) longitudinal optical (LO) phonon scattering. The Fermi levels were 0.1, 0.2, 0.3, 0.4, 0.5, 0.6, 0.8, and 1.0 eV, respectively. (**d**) The influences of several kinds of scattering mechanisms versus temperatures. The Fermi level was 0.5 eV.

Phonons, i.e., the quanta of crystal lattice vibrations, affected the electrical and optical properties of the graphene layer significantly. For instance, in semiconductors, acoustic and optical phonons limited electron mobility and determined their optical response. The main scattering mechanisms of the graphene layer included the collisions of electrons with acoustic phonons (AC), LO phonons and impurity. The AC phonon scattering came from the interactions of electrons with lattice vibrations, representing all atoms in a unit cell moving in the same direction with a small phase difference. The low-energy AC phonon scattering can be treated under elastic scattering approximation. As temperature increased, the influences of AC phonon scattering increased linearly with temperature, as give in Equation (6), which has also been confirmed by supported and suspend graphene samples. For example, if the Fermi level was 0.5 eV, when the temperatures were 300, 500 and 800 K, the AC phonon scattering rates were 1.935 × 10^12^, 3.225 × 10^12^, and 5.160 × 10^12^, respectively. Figure 1a also illustrates the AC phonon scattering rates increased with Fermi level. For example, at a temperature of 500 K, the scattering rates were 6.450 × 10^11^, 1.290 × 10^12^, 3.225 × 10^12^, and 6.450 × 10^12^ when the Fermi levels were 0.1 eV, 0.2 eV, 0.5 eV, and 1.0 eV, respectively. The influences of impurity scattering can be found in Figure 1b, which varied slowly with temperature. The doping concentrations increased with the Fermi level and the carrier screening effects were enhanced, reducing the impurity scattering rates. The results for the LO phonon scattering rates as a function of temperature can be found in Figure 1c. As temperature increased, the LO phonon scattering rates increased, especially at high temperature. For example, if the Fermi level was 1.0 eV, when the temperatures were 300, 500, and 800 K, the scattering rates were 1.251 × 10^11^, 2.632 × 10^12^, and 1.530 × 10^13^, respectively. Additionally, with the increase in Fermi level, the density of states increased and provided much more phase space for phonon scatterings, which resulted into the scattering rates increasing. For instance, at a temperature of 500 K, the scattering rates were 7.272 × 10^11^, 9.076 × 10^11^, 1.528 × 10^12^, and 2.614 × 10^12^ when the Fermi levels were 0.1 eV, 0.2 eV, 0.5 eV, and 1.0 eV, respectively. Thus, the scattering time reduced more significantly at a larger Fermi level (>0.8 eV), resulting in the graphene conductivity reducing. This was different from the results that the scattering time was constant, in which the graphene conductivity increased with Fermi level monotonously. The total scattering rates were associated with AC phonon scattering, impurity scattering, and LO phonon scattering, which was summarized in Figure 1d. The graphene Fermi level was 0.5 eV. At *T* = 300 K the scattering rates of AC phonon, impurity, LO phonon and total scatterings were 1.935 × 10^12^, 2.308 × 10^11^, 7.312 × 10^10^, and 2.239 × 10^12^, respectively. This implied that electrons interacted much more strongly with AC phonons, which dominated at room temperature. For the LO phonon scattering, it increased with temperature drastically and played the dominant role at higher temperature was beyond 600 K. For example, at *T* = 800 K, the scattering rates of AC phonon, impurity, LO phonons and total scatterings were 5.160 × 10^12^, 2.304 × 10^11^, 9.042 × 10^12^, and 1.443 × 10^13^, respectively.

**Figure 2:**
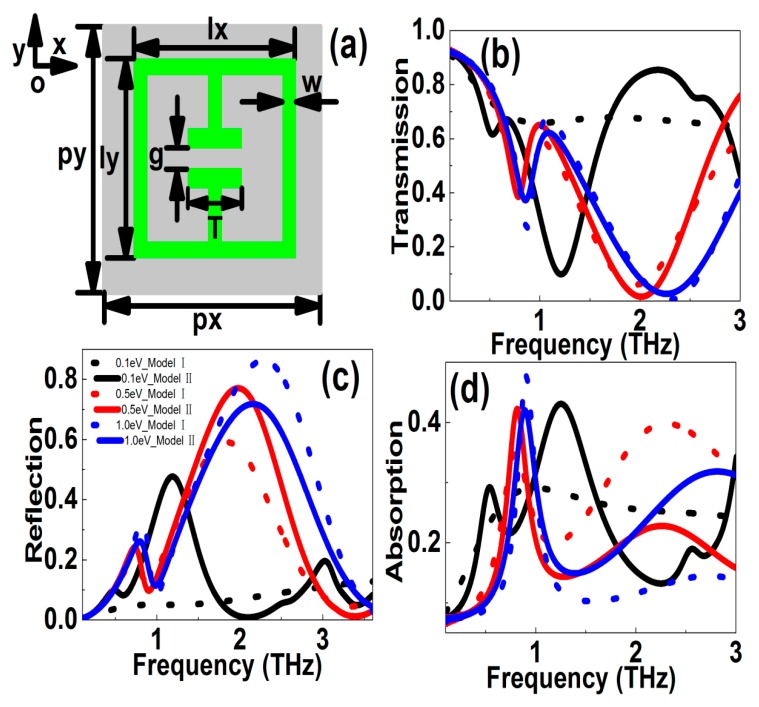
(**a**) Sketches of graphene eSRRs patterns. (**b**–**d**) The transmission, reflection, absorption resonant curves versus frequency at different Fermi levels, respectively. The polarization was along the *y* direction. The Fermi levels were 0.1, 0.2, 0.3, 0.5, 0.8, and 1.0 eV, respectively.

To compare the simulation results obtained from Model I (the scattering time was a constant) and Model II (including the effects of phonon scatterings), we gave the simulation results of the electric split ring resonators (eSRRs) graphene patterns, as shown in Figure 2. The structure parameters were shown in the following, *l_x_* = *l*_y_ = 36 μm, *g* = 6 μm, *w* = 8 μm, and *T* = 18 μm. The polarization was along the *y* direction. Figure 2b shows the transmission curves of the eSRRs graphene patterns. For Model I, i.e., the relaxation time *τ* was a constant and 0.5 *ps* in the manuscript. If the Fermi level was low, e.g., *E*_f_ = 0.1 eV, only dipolar resonances appeared. As the Fermi level increased, the graphene conductivity and permittivity were enhanced, the dynamic kinetic decreased, the dipolar resonance became stronger, and the resonant dip indicated a blue shift. For instance, when the Fermi level changed in the range of 0.1–1.0 eV, the resonant frequency can be modulated in the range of 1.451–2.308 THz, and the transmission dip varied in the range of 0.2988–0.0053. Correspondingly, the frequency and amplitude modulation depths were 37.15% and 98.23%, respectively. Next, we discussed the case of Model II, i.e., the effects of phonon scattering were taken into account. For the high frequency dipolar resonance, the resonant strength increased with Fermi level. But because the effects of AC and LO phonon scatterings dominated when the carrier concentration were large enough, the resonant strength of transmission curves showed a peak at an optimum Fermi level (in the range 0.5–0.8 eV). When the Fermi level changed in the range 0.1–1.0 eV, the resonant frequency can be tailored in the range 1.213–2.263 THz, and the transmission dip modulated in the range 0.01346–0.09844. Correspondingly, the frequency and amplitude modulation depths (MD) were 46.40% and 73.19%, respectively. The strongest resonant curves appeared at the Fermi level of 0.8 eV. This was quite different from Model I, in which the resonances increased with the Fermi level monotonously. Figure 2c shows the reflection curves versus frequency. If the Fermi level changed in the range 0.1–1.0 eV, the resonant frequency of reflection curves was modulated in the range 1.325–2.288 THz, and the reflection peak was tuned in the range 0.2272–0.8633. Correspondingly, the frequency and amplitude MDs were 42.08% and 73.68%, respectively. In Model II, because at a large Fermi level the influences of AC and LO phonon scatterings increased, the resonant strength reduced. For instance, when the Fermi level changed in the range 0.1–1.0 eV, the resonant frequency can be tuned in the range 1.189–2.190 THz, and the reflection peak modulated in the range 0.4768–0.7878. The frequency and amplitude modulation depths were 45.72% and 39.48%, respectively. In addition, Figure 2d shows the simulation results for the absorption curves. When the Fermi level changed in the range 0.1–1.0 eV, the frequency and amplitude modulation depths were 63.53% and 70.23%, respectively. While in Model II, if the effects of phonon scattering were taken into account, the resonant strength shrunk at larger Fermi levels due to the AC and LO phonon scatterings. For instance, when the Fermi level changed in the range 0.1–1.0 eV, the resonant frequency can be tuned in the range 1.252–2.820 THz, and the absorption peak modulated in the scope of 0.2194–0.4746. Correspondingly, the frequency and amplitude modulation depths were 55.61% and 53.77%, respectively. From the above discussions, we can come to the points that if the phonon scatterings were taken into account, the tunable properties of graphene MMs decreased. Finally, it was known to us that graphene plasmons were classified into center waveguide plasmons and edge plasmons. For the large area of 2D graphene patterns (which means that a graphene sheet is infinite in extent), waveguide plasmons dominated, whose fields concentrated within the center area. When the width of a graphene ribbon was reduced to a few tens of nanometers, the waveguide mode disappeared and only the edge modes were left. For our proposed subwavelength THz graphene patterns, the structural parameters were relatively large, in the range of tens of micrometers, and the effects of edge property were not very significant and can be omitted.

**Figure 3:**
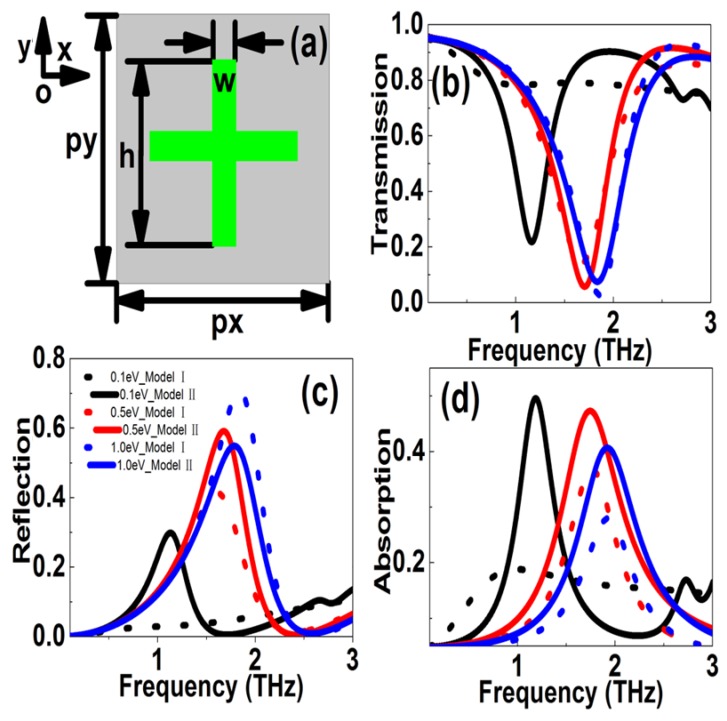
(**a**) is the sketch of the graphene cross patterns. (**b–d**) The transmission, reflection, absorption resonant curves versus frequency at different Fermi levels. The Fermi levels were 0.1, 0.5, and 1.0 eV, respectively. The stripe length and width were 48 and 8 μm, respectively. The periodic lengths along *x* and *y* directions were both 72 μm.

Figure 3 shows the simulation results of the graphene MM cross patterns. The top view of the graphene cross ribbon structures can be found in Figure 3a. The periodic lengths along *x* and *y* directions were *p*_x_ and *p*_y_ with the values of 72 μm. The length and width of cross graphene patterns were 48 μm and 8 μm, respectively. The polarization was along the *y* direction. Figure 3b is the transmission response curves versus frequency. The Fermi levels were 0.1, 0.5, and 1.0 eV, respectively. For Model I, the value of conductivity and graphene permittivity increased with the Fermi level, and the graphene indicated a strong transmission response. For instance, when the Fermi level changed in the range 0.2–1.0 eV, the resonant frequency modulated in the range 1.349–1.860 THz, and the transmission dip can be tuned in the range 0.4791–0.02665. Correspondingly, the frequency and amplitude MDs were 27.47% and 94.44%. If the LO phonon and impurity scatterings were taken into account in Model II, the scattering procession was inelastic, which meant that the relaxation time of the carrier was also related to mobility and the Fermi level. If the *E*_f_ was low, the relaxation time was relatively larger, leading to higher graphene conductivity and permittivity. As the Fermi level increased, the relaxation time increased. But if the Fermi level increased further, the scattering of AC phonon and LO phonons enhanced, resulting in the relaxation time decreasing. When the Fermi level was 0.5 eV, the resonant strength was the strongest. The strongest resonant dip was not coinciding with the largest Fermi level, different from the results obtained from the original Model I. The resonant properties showed a peak with the increase in Fermi level, the optimum value of *E*_f_ was about 0.5–0.8 eV. In this case, when the Fermi level changed in the range 0.1–1.0 eV, the resonant frequency modulated in the range 1.164–1.836 THz, and the transmission dip can be tuned in the scope of 0.2173–0.02351. Correspondingly, the frequency and amplitude modulation depths were 36.60% and 89.18%, respectively. 

Figure 3c shows the reflection resonant curves for cross graphene patterns. In Model I, if the Fermi level changed in the range 0.2–1.0 eV, the frequency and amplitude modulation depths were 33.68% and 82.74%, respectively. While if the phonon scattering rates were taken into account, as the Fermi level increased, the graphene conductivity and permittivity showed a peak because the AC and LO phonon scattering increased at larger Fermi levels. In this case the reflection peak value showed a maximum at 0.8 eV. When the Fermi level changed in the range 0.1–1.0 eV, the resonant frequency can be changed in the range 1.132–1.790 THz, and the reflection peak modulated in the range 0.2985–0.6077. Correspondingly, the frequency modulation depth was 36.75%, while the amplitude modulation depth was 50.88%. The simulation results for the absorption curves can be found in Figure 3d. At large Fermi levels, e.g., 1.0 eV, because the AC and LO phonon scatterings increased drastically, the results obtained from Model II was larger than those acquired from the more conventional Model I.

**Figure 4:**
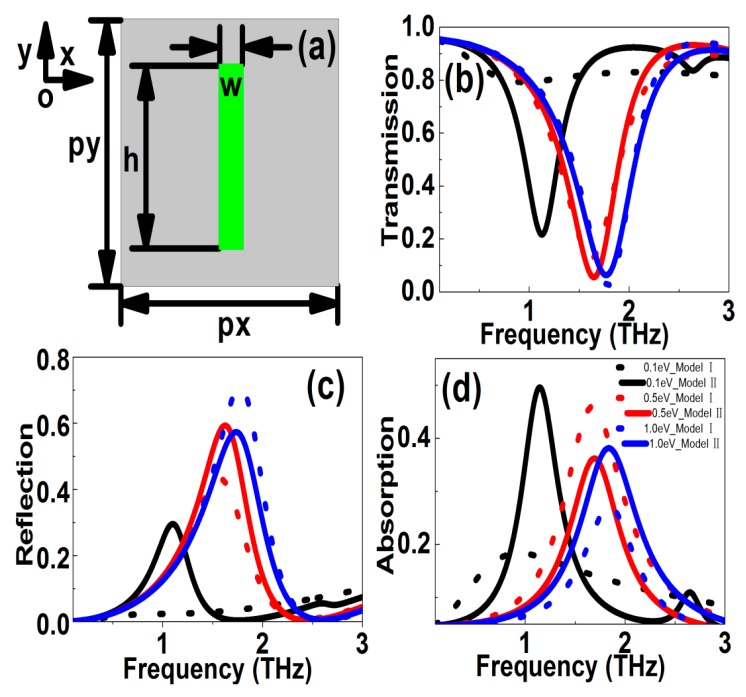
(**a**) Sketch of graphene cut wire patterns. (**b–d**) The transmission, reflection, absorption resonant curves versus frequency at different models. The Fermi levels were 0.1, 0.2, 0.3, 0.4, 0.5, 0.6, 0.8, and 1.0 eV, respectively. The stripe length and width were 48 μm and 8 μm, respectively. The periodic lengths along *x* and *y* directions were both 72 μm.

Figure 4 illustrates the influences of Fermi levels on the resonant curves of graphene cut wire. The stripe length and width were 48 μm and 8 μm, respectively. The periodic lengths along *x* and *y* directions were both 72 μm. It can be found from Figure 4a that for the symmetric graphene ribbon structure, an obvious resonant dip was observed, resulting from the dipolar resonances. With the graphene Fermi level increased, the resonant strength became stronger, and the resonant peak shifted to a larger frequency, i.e., blue shift. In Model I, if the values of *E*_f_ were 0.2, 0.5, and 1.0 eV, the resonant frequencies were 1.315, 1.619, and 1.791 THz, and the values of resonant dips were 0.4936, 0.1266, and 0.02555, respectively. Accordingly, the frequency and amplitude modulation depths were 26.58% and 94.82%. On the other hand, in Model II, if the phonon scattering was taken into account, when the *E*_f_ changed in the range 0.1–1.0 eV, the frequency and amplitude modulation depths were 21.16% and 35.45%, respectively. Usually the tunable properties of graphene MM structures reduced if the phonon scattering was included. Another point should be noted was that with the increase of Fermi level, the resonant curve became stronger and sharper with a large value of *Q*-factor. For instance, In Model I, if the graphene Fermi levels were 0.1, 0.2, 0.5, and 1.0 eV, the *Q*-factors of resonant curves were 1.334, 1.591, 2.514, and 2.940. The values of FOM were 0.05021, 0.5831, 1.936, and 2.693, respectively. On the other hand, for the case of Model II, if the graphene Fermi levels were 0.1, 0.2, 0.5, and 1.0 eV, the *Q*-factors of resonant curves were 2.748, 2.909, 2.835, 2.718, and the values of FOM were 1.945, 2.426, 2.487, and 2.314, respectively. As Fermi level increased, the graphene layer showed better plasmonic properties, but at the same time, the dissipation losses also improved. Consequently, the value of the *Q*-factor and FOM showed a peak. 

**Figure 5:**
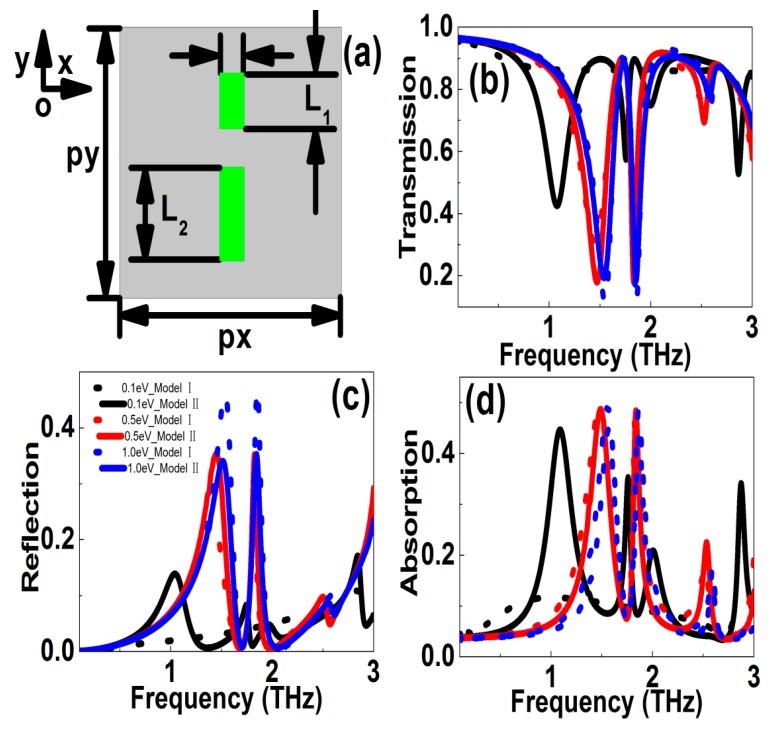
(**a**) is the sketch of the asymmetrical graphene stripe pattern. (**b**–**d**) The transmission, reflection, absorption resonant curves versus frequency, respectively. The Fermi levels were 0.1, 0.5, and 1.0 eV, respectively. The asymmetrical degree *δ* was *δ* = *L*_2_ − *L*_1_. The stripe length and width were 96 μm and 8 μm, respectively. The periodic lengths along *x* and *y* directions were both 108 μm.

The plasmonic induced transparency (PIT) phenomenon can be obtained by breaking the structural symmetry or subwavelength-scale coupling. Figure 5 illustrates the influences of an asymmetrical degree on the resonant curves of graphene cut wire patterns. The graphene Fermi levels were 0.1, 0.5, and 1.0 eV, respectively. The asymmetrical degree δ was δ = *L*_2_ − *L*_1_. The stripe length and width were 96 μm and 8 μm, respectively. The periodic lengths along *x* and *y* directions were both 108 μm. It can be found from Figure 5a that for the asymmetric graphene stripe structure, i.e., the length of the upper section was smaller than that of the bottom section, an obvious peak was observed. With the graphene Fermi level increased, Fano resonance strength became stronger, and the resonant peak shifted larger frequency, i.e., blue shift. In Model I, the values of *E*_f_ were 0.2, 0.5, and 1.0 eV, the resonant frequencies were 1.696, 1.728, and 1.728 THz, and the values of Fano resonant peaks were 0.8163, 0.8778, and 0.9036, respectively. On the other hand, for the case of Model II, the phonon scattering was taken into consideration, and if the values of *E*_f_ were 0.2, 0.5, and 1.0 eV, the resonant frequencies were 1.678, 1.717, and 1.735 THz, and the values of Fano resonant peaks were 0.9072, 0.9010, and 0.8881, respectively. Figure 5b shows that a novel peak appeared in the reflection curves because of Fano resonance, which also increased with the graphene Fermi level. The effects of the graphene Fermi level on the absorption curves can also be found in Figure 5c, indicating a novel resonant peak near the Fano resonance. Another point to be noted was that with the increase in Fermi level, the Fano resonant curve became stronger and sharper with a large *Q*-factor value. For instance, if phonon scattering was taken into account in Model II, when the graphene Fermi levels were 0.1, 0.5, and 1.0 eV, the *Q*-factors of the resonant curves were 3.113, 7.291, and 6.112, and the values of FOM were 1.005, 5.264, and 4.247, respectively. As the Fermi level increased, the graphene layer showed better plasmonic properties, the carrier concentration also increased, and the dissipation losses improved. Consequently, the values of the *Q*-factor and FOM showed a peak.

**Figure 6:**
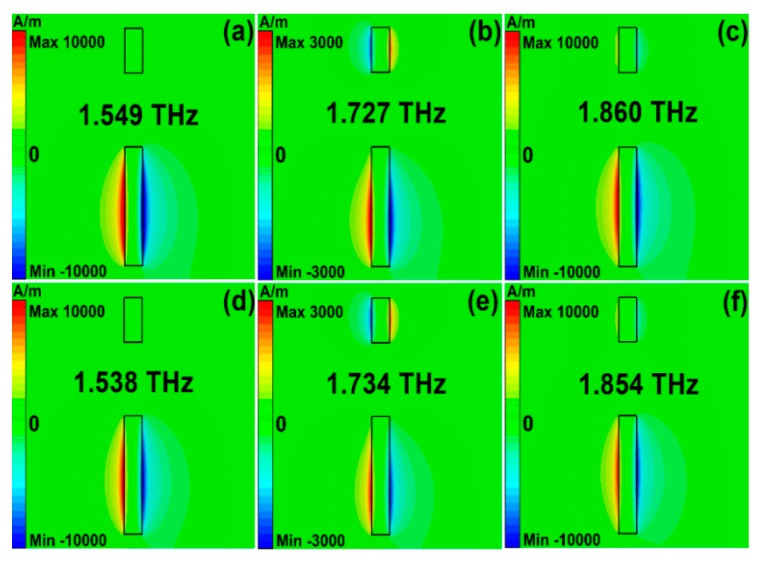
The magnetic fields for the asymmetrical graphene cut wire patters structures. (**a**–**c**) The simulation results for Model I. (**d**–**f**) The simulation results for the phonon scattering was taken into account. The polarization direction of incident light was along the *y* direction.

**Figure 7:**
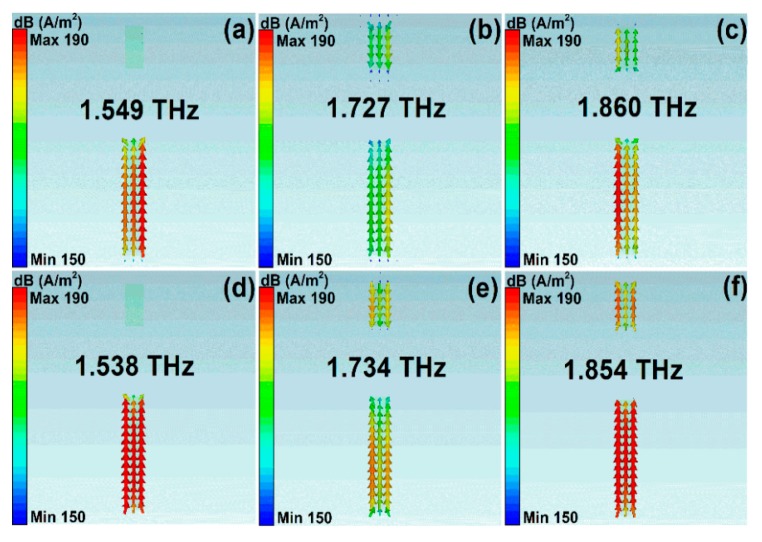
The surface current density for the graphene cut wire stripe patters. (**a–c**) The simulation results for Model I. (**d–f**) The simulation results on condition that the phonon scattering was taken into account. The polarization direction of incident light was along the *y* direction.

2D field plots are good means to understand the mechanisms of resonant spectral curves. To have a deep understanding of different scattering mechanisms on the propagation properties, we demonstrated the surface current density and magnetic fields along the *z* direction at resonant peaks. Figure 6 and Figure 7 show the values of magnetic fields (H_z_) and surface current density (SCD) of asymmetrical stripe MM structures. The polarization of incident light was along the *y* direction. The asymmetrical degree was 30 μm. The Fermi level was 1.0 eV. In Model I, the resonant frequencies were 1.549 THz, 1.727 THz, and 1.860 THz, as shown in Figure 6a–c and Figure 7a–c. At the low frequency of 1.549 THz, the electric dipolar mode of the bottom section of the asymmetrical stripe was excited and behaved as a bright mode. At the Fano frequency of 1.727 THz, the bottom section and the whole stripe interacted, resulting in a net dipole moment. Consequently, a quadrupole-like mode was excited with very low radiation losses. The anti-phase resonance of the quadrupolar mode interacted with the original electric dipolar resonance strongly, resulting in a Fano resonant peak. While in Model II, if the phonon scatterings were taken into account, the resonant frequencies were 1.538 THz, 1.734 THz, and 1.854 THz, respectively, which can be found Figure 6d–f and Figure 7d–f. At the Fano frequency of 1.734 THz, the quadrupolar-like mode interacted strongly with the original electric dipolar resonance and led to a Fano resonant peak. Compared with Figure 6a–c in Model I, the resonant strength of the Fano resonance became weak, which resulted from the large losses if phonon scattering was taken into account.

**Figure 8:**
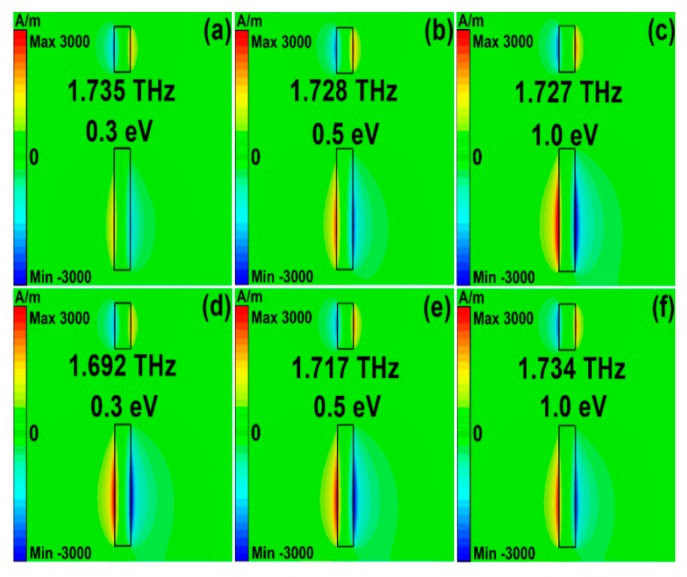
The magnetic fields for the asymmetrical graphene stripe patters structures at different Fermi levels. (**a–c**) The simulation results for Model I. (**d–f**) The simulation results for Model II on condition that the phonon scattering was taken into account. The polarization direction of incident light was along the *y* direction. The graphene Fermi levels were 0.3, 0.5, and 1.0 eV, respectively.

**Figure 9:**
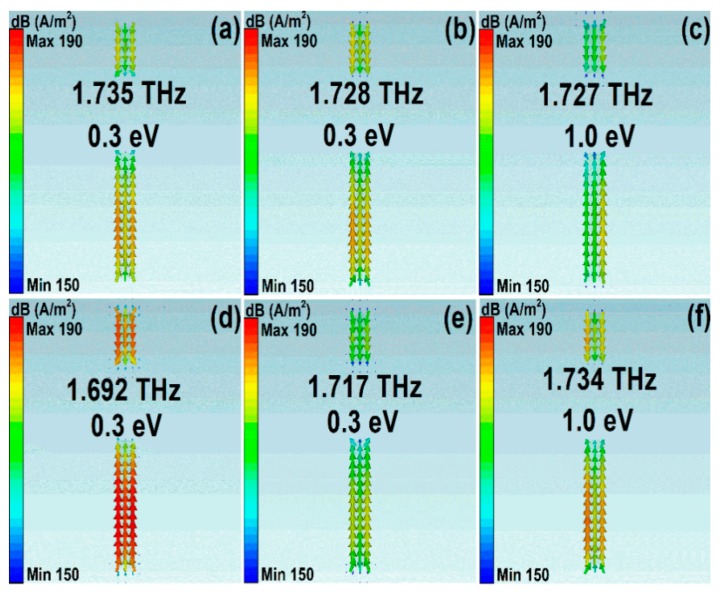
The surface current density for the asymmetrical graphene stripe patters structures at different Fermi levels. (**a–c**) The simulation results for Model II. (**d–f**) The simulation results for Model II on condition that the phonon scattering was included. The polarization direction of incident light was along the *y* direction. The graphene Fermi levels were 0.3, 0.5, and 1.0 eV, respectively.

Figure 8 and Figure 9 show the magnetic fields and surface current density of asymmetrical stripe MM structures at different Fermi levels. The Fano resonant frequencies were 1.735, 1.728, and 1.727 THz, with the Fermi levels being 0.3, 0.5, and 1.0 eV, respectively. The polarization direction of incident light was along the *y* direction. The asymmetrical degree was 30 μm. It can be found from Figure 8a–c and Figure 9d–f that at the Fano resonances both the bottom and upper sections were excited. As Fermi levels increased, the carrier concentration increased, the graphene layer showed better plasmonic properties, and the interaction between the bottom and upper sections were stronger, which can be found in Figure 8 and Figure 9. While in Model II, if the phonon scattering was taken into account, the resonant frequencies were 1.692 THz, 1.717 THz, and 1.734 THz, respectively. Compared with Figure 9a–c, the resonant strengths of the Fano resonances in Model II were weaker, resulting from the large losses in phonon scattering.

## 4. Conclusions

The influences of phonon scattering mechanisms on the tunable propagation properties of several kinds of graphene patterns structures have been thoroughly discussed, also including the effects of graphene pattern structures (eSRRs, cross, and cut wire stripes), Fermi levels, and operation frequencies. The results showed that, at room temperature, the AC phonon scattering dominated, and the effects of impurity scattering can be omitted. As temperature increased, LO phonon scattering increased and played a dominant role if temperature went beyond 600 K. In addition, as Fermi level increased, the effects of AC and LO phonon scatterings increased and the impurity scattering reduced. In our proposed Model II, if the phonon scattering was taken into account, because the AC and LO phonon scatterings increased significantly at larger Fermi levels, the resonant properties showed a peak at an optimum Fermi level (0.5–0.8 eV). This was different from the results of the constant scattering time approximation, where the resonant strength increased with graphene Fermi levels. In addition, the tunable properties of the graphene MM structures were reduced at larger Femi levels because of phonon scatterings. The results are very helpful to further understand the importance of graphene electronic properties (carrier screening, scattering, and energy dissipation) and to design novel tunable functional devices, e.g., sensors, modulators, and antennas. 

## Supplementary Materials

The following are available online at https://www.mdpi.com/2079-4991/10/1/39/s1, The supplementary file shows the fabrication, transfer, and characterization of graphene metamaterials structures.
