# Supplementary material for: Investigation of Phonon Scattering on the Tunable Mechanisms of Terahertz Graphene Metamaterials
Source: Nanomaterials (Basel). 2019 Dec 23;10(1):39. doi: 10.3390/nano10010039 (PMC7023326; doi:10.3390/nano10010039)
Supplement: Supplementary file 1 [file nanomaterials-10-00039-s001.pdf]

Supplementary Materials

Investigation of Phonon Scattering on the Tunable Mechanisms of Terahertz Graphene Metamaterials

Xiaoyong He [1,2,*], Fangting Lin [1,2], Feng Liu [1,2] and Hao Zhang [1]

1. Department of Physics, Mathematics & Science College, Shanghai Normal University, No. 100 Guilin Road, Shanghai 200234, China; nounou7@163.com (F.L.); fliu@shnu.edu.cn (F.L.); Zhanghao@shnu.edu.cn (H.Z.)
2. Shanghai Key Lab for Astrophysics, No. 100 Guilin Road, Shanghai, 200234, China

* Correspondence: xyhethz@hotmail.com (X.H.); Tel.: +86-13661584901

I. The Fabrication of Graphene Layer

The fabrication of large area monolayer graphene and integration with silicon devices are important to design tunable terahertz graphene metamaterials devices. High quality uniform graphene layer can be obtained from the CVD method deposition on the metal substrate. A typical example of growth process was given below. The graphene films were primarily grown on 25-μm thick Cu foils in a hot wall furnace consisting of a fused silica tube. (i) The fused silica tube with the Cu foil was loaded, evacuated, back fill with hydrogen, heat to 1000 °C and maintain a H_2 (g) pressure of 40 mTorr under a 2 sccm flow; (ii) The Cu film at the desired temperatures was stabilized, up to 1000 °C, and introduce 35 sccm of CH_4 for a desired period of time at a total pressure of 500 mTorr; (iii) after exposure to CH_4, the furnace was cooled to room temperature. The heating rate was about 20 °C/min, and the cooling rate varied in the range of 40–300 °C/min. The heating temperature was about 1000 °C, and the keeping time was about 30 min [S1–S4].

II. The Transfer of Graphene Layer

Graphene films were removed from the Cu foils by etching in an aqueous solution of iron nitrate. The etching time was associated with the etchant concentration, the area, and the thickness of Cu foils. Typically, a 1 cm^2 by 25-μm thick Cu foil can be dissolved by a 0.05 g/mL iron nitrate solution over nights. There were two methods to transfer the graphene from the Cu foils: (i) After the copper film was dissolved, a substrate was brought into contact with the graphene film and it was ‘pulled’ from the solution; (ii) The surface of the graphene-on-Cu was coated with polydimethylsiloxane (PDMS) or poly-methyl methacrylate (PMMA) with a thickness about 100 nm, followed by etching of the copper and catalysts in $FeCl_3$ solution. PMMA-supported graphene films were then thoroughly rinsed in deionized water several times to rinse the etchant residue and then scooped by the substrate. Then, the chip was dried in air overnight, the sample was submerged in acetone to remove the PMMA. Then, the as-prepared samples were dried carefully under a gentle stream of N_2 gas, and finally the graphene layers were transferred onto the desired substrates such as SiO_2/Si multilayers before storage [S5,S6].

III. The Fabrication of Graphene Subwavelength Patterns

The transferred graphene layer was typically *p*-doped. The designed graphene unit cell arrays patterns can be fabricated by using standard optical lithography or e-beam lithography with poly-methyl methacrylate as an electron beam resist. Oxygen plasma can be used to etch away the exposed area, leaving the periodic pattern of graphene patterns protected by a PMMA layer, which was then removed with acetone. Next, the ion-gel layer spin-coated onto the graphene pattern to yield a high capacitance (C = 2.45 μF/cm^2) [S7]. To prepare the ion gel, the liquid ([EMIM][TFSI]) was first dried under vacuum and then it was dissolved with 22 mg of PS-PEO-PS (10–44–10 kg·mol^{-1}) triblock copolymer in 2 mL of dry dichloromethane. Finally, in order to tune and monitor the Fermi level of graphene layer in situ, a Ti/Au (the thickness was about tens of nm, e.g. 10/100 nm) electrode

as a top gate deposited onto the ion-gel layer through electron-beam lithography and a lift-off process, and the doped Si layer acted as a back gate electrode. The ion-gel layer also served as a transparent spacer layer between the top Au contact and the bottom layer of graphene nanostructures. In this way, the graphene Fermi level could be effectively tuned in a wide range (0.10–1.0 eV) by applying a top gate bias voltage of 0.2–2.3 V. The Fermi energy was determined through $E_F = \hbar v_F(\pi n)^{1/2}$, where $v_F = 10^6$ m/s is the Fermi velocity, and the carrier density n is calculated by $n = C\Delta V/e$, where ΔV is the gate bias [S8,S9].

IV. The Characterization of Graphene Devices

The characteristics of graphene samples can be obtained from SEM and TEM images, revealing the Cu grains clearly. Raman spectroscopy offered a powerful tool to probe the structural characteristics and properties of graphene. The thickness, quality, uniformity, and different layer numbers graphene, can be distinguishable and evaluated in Raman spectroscopy. For instance, the quality of CVD-grown graphene can be characterized by Raman spectroscopy, where a negligible D-band was observed—indicating a low defect density—and a 2D to G ratio of two confirmed the graphene monolayer sheet. The Raman spectra can also be used to determine and monitor the electron/hole dopants in graphene, e.g. the thermal conductivity and strain of graphene were extracted from the shift of G band and 2D band frequency [S10]. X-ray photoelectron spectroscopy can probe the composition and doping-induced valence band shifts in the annealed and hydrazine-doped samples. The morphology of graphene nanoribbons were characterized by Atom Force Microscopy [S11]. In addition, electrical transport properties at room temperature were characterized with a semiconductor parameter analyzer. The graphene Fermi level position was determined by measuring the gate-dependent sheet resistance and assigning the resistance maximum as the charge neutral point, where the graphene Fermi level was aligned with the Dirac point. After identifying the charge neutral point, a simple capacitor model was used to calculate the graphene Fermi level position for a given gate bias [S12].

References

S1. X. Li, W. Cai, J. An, S. Kim, J. Nah, D. Yang, R. Piner, A. Velamakanni, I. Jung, E. Tutuc, S. K. Banerjee, L. Colombo, and R. S. Ruoff, "Large area synthesis of high-quality and uniform graphene films on copper foils," Science **324**, 1312-1314 (2009).

S2. T. Wu, X. Zhang, Q. Yuan, J. Xue, G. Lu, Z. Liu, H. Wang, H. Wang, F. Ding, Q. Yu, X. Xie, and M. Jiang, "Fast growth of inch-sized single-crystalline graphene from a controlled single nucleus on Cu–Ni alloys," Nat. Mater. **15**, 43-47 (2016).

S3. W. Gao, J. Shu, K. Reichel, D. V. Nickel, X. He, G. Shi, R. Vajtai, P. M. Ajayan, J. Kono, D. M. Mittleman, and Q. Xu, "High-contrast terahertz wave modulation by gated graphene enhanced by extraordinary transmission through ring apertures," Nano Lett. **14**, 1242-1248 (2014).

S4. R. Degl'Innocenti, D. S. Jessop, Y. D. Shah, J. Sibik, J. A. Zeitler, P. R. Kidambi, S. Hofmann, H. E. Beere, and D. A. Ritchie, "Low-bias terahertz amplitude modulator based on split-ring resonators and graphene," ACS Nano **8**, 2548-2554 (2014).

S5. L. Gao, G. X. Ni, Y. Liu, B. Liu, A. H. C. Neto, and K. P. Loh, "Face-to-face transfer of wafer-scale graphene films," Nature **505**, 190-194 (2014).

S6. R. Vishwakarma, M. S. Rosmi, K. Takahashi, Y. Wakamatsu, Y. Yaakob, M. I. Araby, G. Kalita1, M. Kitazawa, and M. Tanemura, "Transfer free graphene growth on SiO_2 substrate at 250 °C," Sci. Rep. **7**, 43756 (2017).

S7. V. W. Brar, M. S. Jang, M. Sherrott, J. J. Lopez, and H. A. Atwater, "Highly confined tunable mid-infrared plasmonics in graphene nanoresonators," Nano Lett. **13**, 2541-2547 (2013).

S8. S. Kim, M. S. Jang, V. W. Brar, Y. Tolstova, K. W. Mauser, and H. A. Atwater, "Electronically tunable extraordinary optical transmission in graphene plasmonic ribbons coupled to subwavelength metallic slit arrays," Nat. Commun. **7**, 12323 (2016).

S9. H. Goto, E. Uesugi, R. Eguchi, A. Fujiwara, and Y. Kubozono "Edge-dependent transport properties in graphene," Nano Lett. **13**, 1126-1130 (2013).

S10. J. Chen, M. Badioli, P. Alonso-Gonzalez, S. Thongrattanasiri, F. Huth, J. Osmond, M. Spasenovic, A. Centeno, A. Pesquera, P. Godignon, A. Zurutuza Elorza, N. Camara, F. J. Garcia de Abajo, R. Hillenbrand, and F. H. L. Koppens, "Optical nano-imaging of gate-tunable graphene plasmons," Nature **487**, 77-81 (2012).

S11. B. Sensale-Rodriguez, R. Yan, M. M. Kelly, T. Fang, K. Tahy, W. S. Hwang, D. Jena, L. Liu, and H. G. Xing, "Broadband graphene terahertz modulators enabled by intraband transitions," Nat. Commun. **3**, 378 (2012).
S12. J. B. Bult, R. Crisp, C. L. Perkins, and J. L. Blackburn, "Role of dopants in long-range charge carrier transport for p-type and n-type graphene transparent conducting thin films," ACS Nano **7**, 7251-7261 (2013).
